# SARS-CoV-2 infection during pregnancy: clinical characteristics and vertical transmission in a referral hospital in Peru

**DOI:** 10.17843/rpmesp.2024.412.13293

**Published:** 2024-05-20

**Authors:** Claudia Aracelli Urbina-Alvarez, Julio Cesar Sifuentes-Alvarez, Juan Felipe Moreno-Bocanegra, Kevin Vasquez-Sandoval, Lilia Huiza-Espinoza, Mauricio La Rosa-De los Rios, Juan Carlos Gomez De La Torre-Pretell, Claudia Fiorella Barletta-Carrillo

**Affiliations:** 1 Edgardo Rebagliati Martins National Hospital, Lima, Peru. Hospital Nacional Edgardo Rebagliati Martins Lima Peru; 2 Hospital San Bartolomé, Lima, Peru. Hospital San Bartolomé Lima Peru; 3 University of Texas Medical Branch, Texas, United States. University of Texas Universidad De Texas Medical Branch Texas USA; 4 Universidad Peruana Cayetano Heredia, Lima, Peru. Universidad Peruana Cayetano Heredia Universidad Peruana Cayetano Heredia Lima Peru; 5 Laboratorio ROE, Lima, Peru. Laboratorio ROE Lima Peru; 6 Sequence Reference Lab, Lima, Peru. Sequence Reference Lab Lima Peru; 7 Universidad Nacional Mayor de San Marcos, Lima, Peru. Universidad Nacional Mayor de San Marcos Universidad Nacional Mayor de San Marcos Lima Peru

**Keywords:** Infectious disease transmission, vertical, COVID-19, Pregnancy, Newborn

## Abstract

The aim of this study was to analyze the vertical transmission of SARS-CoV-2 in pregnant women with COVID-19 in the Gynecology and Obstetrics Department of the Edgardo Rebagliati Martins National Hospital (HNERM). Twelve pregnant women who met the inclusion criteria were included. Real-time PCR (RT-PCR) tests for SARS-CoV-2 were performed when each woman was admitted to the hospital, placenta samples were collected for pathological evaluation as well. The results showed that vertical transmission of the virus was rare, with an overall low positivity rate in newborns. Although the study has limitations, such as the small number of cases and the lack of electron microscope analysis, it is the first attempt to evaluate vertical transmission in Peru. It is concluded that more research is needed to better understand the relationship between COVID-19 infection and complications during pregnancy.

## INTRODUCTION

The COVID-19 pandemic continued to exert a significant impact worldwide during the first week of February 2022, with a total of 490 million confirmed cases and more than 5.8 million deaths. Peru was one of the countries most affected by this disease, with more than 3.3 million confirmed cases and almost 207,000 deaths ^1^. Several studies have shown that SARS-CoV-2 infection can cause multisystemic involvement in pregnant women, with complications ranging from premature birth to maternal death ^2^. In Peru, as in other parts of the world, COVID-19 has had a significant impact on maternal health care. There was a 12% increase in maternal deaths during 2021 compared with 2020, and one in nine of these deaths was related to COVID-19 ^3^. The most common complications were premature rupture of membranes and preeclampsia, while 14% of newborns presented conditions such as prematurity, low birth weight, sepsis, and pneumonia ^4^.

During pregnancy, physiological changes occur that make pregnant women an at-risk population ^5^^,^^6^. COVID-19 can have serious repercussions in pregnant women. In addition, although vertical transmission of coronavirus is a rare event ^7^, problems related to this transmission are emerging ^8^. Viremia was present in 1% of adult patients showing symptoms of coronavirus disease in 2019 ^9^. Perinatal exposure, type of delivery, and time from delivery to diagnosis of neonatal infection are considered to determine whether infection occurred congenitally or perinatally. Newborns with coronavirus infection are usually asymptomatic ^9^.

There is very little evidence of intrauterine transmission from mother to fetus or intrapartum transmission from mother to newborn. In cases of late maternal infection, the possibility that the newborn is actively infected and thus at risk for complications should be considered, as well as the possibility that the infant poses risks to health care workers. In this article, we focused on newborns whose mothers had documented or suspected COVID-19 at the time of delivery ^6^^,^^10^^,^^11^. Therefore, this study aimed to analyze the vertical transmission of SARS-CoV-2 in pregnant women with COVID-19 at the Edgardo Rebagliati Martins National Hospital (HNERM).

**KEY MESSAGES**

Motivation for the study: There is a gap in knowledge about vertical transmission of SARS- CoV-2 and its implications for maternal and neonatal health, despite evidence of multisystem involvement in pregnant women with COVID-19.

Main findings: The study results suggest low incidence of vertical transmission during pregnancy, with only one PCR-positive case in the placenta and one asymptomatic neonate.

Implications: Our results can inform strategies for prevention and management of COVID-19 in pregnant women, as well as guide the development of health policies aimed at protecting maternal and neonatal health during the pandemic.

## THE STUDY

### Design and setting

This was a descriptive study that was conducted in the Department of Gynecology and Obstetrics at HNERM between January 2021 and January 2022.

### Population

The study included pregnant women admitted to HNERM who met the following inclusion criteria: clinical and/or radiological and/or laboratory suspicion of SARS-CoV-2 infection and any indication for termination of pregnancy by cesarean section in the second and third trimester. Participants signed an informed consent form. Patients who had had an abortion and/or were in the first trimester of gestation were excluded from the study, as well as those who did not give their consent to participate. Twelve pregnant women met the selection criteria.

### Study variables

The independent variable was the degree of severity of COVID-19 infection during pregnancy; cases were classified as proposed in Wuhan: moderate (mild pneumonia without acute respiratory failure or inflammatory response), severe (pneumonia with acute respiratory failure, inflammation or hypercoagulability) and critical (with criteria for intubation and invasive ventilation, shock or multiorgan failure).

The study variables, their categories and units of measurement are shown in Table 1. Data were collected on a single chart for each patient at a single time interval for subsequent analysis.

**Table 1:** Maternal characteristics of patients included in the study (n=12).

Variable		Unit of measurement
Sociodemographic characteristics		
	Maternal age	years
	History of mean gestational age at delivery	weeks
	Neonatal mortality	yes/no
Clinical findings		
	HR	beats/min
	RR	breaths/min
	SBP	mmHg
	DBP	mmHg
	Oxygen saturation	%
	Oxygen requirement	L
	Radiological findings of pneumonia	yes/no
	Admission to maternal ICU	yes/no
	Use of mechanical ventilation	yes/no
	Time of illness	days
Laboratory findings		
	LDH	U/L
	CRP	U/L
	TGO	U/L
	Platelet count	per 1000/UL
	Leucocytes	per 1000/UL
	Creatinine	mg/dL
	Lymphocytes	per 1000/UL

HR: heart rate, RR: respiratory rate, SBP: systolic blood pressure, DBP: diastolic blood pressure, ICU: intensive care unit, LDH: lactate dehydrogenase, CRP: C-reactive protein, TGO: oxaloacetic transaminase.

### Data source/measurement and procedure

Real-time PCR (RT-PCR) diagnostic testing for SARS-CoV-2 was performed at admission of each pregnant woman by obtaining nasal swab samples from the pregnant woman, nasal swab samples from the neonate within 72 hours, placental membrane, amniotic fluid, and cord blood swab samples. Placenta samples were obtained for later microscopic evaluation.

For membrane swabbing and cord blood collection, placental swabs (placental PCR) were obtained from the amniotic (fetal) surface after clearing the surface of maternal blood and were collected from the space between the amnion and chorion after careful manual separation of the membranes. For cord blood samples, plasma was extracted and stored at -80 °C until processing.

PCR testing for SARS-CoV-2 was performed by RNA extraction from biological samples with Sbeadex livestock kit reagent (LGC Biosearch Technologies) on oKtopure automated platform and molecular detection through amplification of the virus RdRp gene and RNAse P as internal control with the Logix Smart SARS-CoV-2 test kit from Co-Diagnostics Inc on Cobas Z 480 (Roche) or CoDx-Box (Biomolecular Systems) thermal cyclers.

Placentas were formalin-fixed and embedded in kerosene, stained with hematoxylin/eosin stain. Immunohistochemistry with CD34 was also performed.

Regarding pathological anatomy, microscopic placental findings were classified as acute inflammatory pathology corresponding to stages of maternal and fetal inflammation with neutrophil infiltrate (histological chorioamnionitis and umbilical arteritis); and chronic inflammatory pathology: lymphocyte and histiocyte infiltrate (chronic lymphocytic villitis and chronic deciduitis with plasma cells). The Amsterdam Consensus ^12^ was used for defining chronic intervillous villitis: low grade (less than 10 villi affected per focus or microscopic view and multifocal if found in several slides); and, high grade (greater than 10 villi in any field, it can be patchy or diffuse, if more than 30% of the parenchyma is affected).

### Statistical analysis

The data were transferred to a MS Excel spreadsheet for coding and entry into the STATA program version 16.0 for Windows for statistical analysis. Since this was a descriptive study, the data were double coded and error corrected. For quantitative variables, normality was evaluated with the Shapiro-Wilk test and the mean and standard deviation or median and interquartile range were used, according to their distribution.

### Ethical aspects

Before starting the study, each participant was asked to sign an informed consent form. The study did not present risks for the patients. Strict biosafety protocols were followed during sample collection, processing and transfer. The study was evaluated and approved by the Research Ethics Committee Specialized in COVID-19 of Essalud. (Resolution N°42-IETSI-ESSALUD 2020 dated March 27, 2020) and PRISA code No. EI00001364.

## FINDINGS

### Maternal-perinatal outcomes

The characteristics of the study participants are presented in Table 2. Of the twelve pregnant women included, eleven were singleton pregnancies and one was a twin. Among the symptoms of the pregnant women, one had odynophagia, two had chest pain, two had nasal flaring, four had fever, seven had dyspnea and eight had cough. Nine had a positive molecular PCR test for SARS-CoV-2 and three were included due to clinical, radiological and antigenic test results compatible with COVID-19. Two cases of SARS-CoV-2 infection were severe and four critical cases were admitted to the intensive care unit (ICU). All patients underwent cesarean section. There was one positive case of SARS-CoV-2 in an asymptomatic neonate (Table 3).

**Table 2:** Maternal characteristics of patients included in the study (n=12).

Characteristics	Mean (min-max)
Maternal age	33.8 (29-42 years)
Gestational age	34:3 (28-39 weeks)
Admission to maternal ICU	6
Pneumonia	6
Mechanic ventilation	4
Time of illness	5.4 (2-15)
LDH	326.2 (183-391)
PCR	504 (11-1735)
TGO	70.5 (28-164)
Platelet count	262 (110-936)
Leucocytes	11.1 (5.3-26.0)
Creatinine	0.5 (0.4-0.8)
Lymphocytes	1.4 (0.5-4.0)
HR	93 (74-118)
RR	22 (17-28)
SBP	113 (100-130)
DBP	67.5 (60-70)
Oxygen saturation	90.4 (63-98)
Oxygen requirement	12.28 (3-16)

ICU: intensive care unit; LDH: lactate dehydrogenase; CRP: C-reactive protein; TGO: oxaloacetic transaminase; SBP: systolic blood pressure; DBP: diastolic blood pressure.

**Table 3:** Characteristics of each case included.

Case	Age of the mother	GA	Maternal PCR	Severity of maternal infection	Placental PCR	Liquid PCR	Cord PCR	Day of life 1	Day of life 2	Day of life 3	Neonatal outcome
Case 1	31	37	Negative	Critical	Positive (Ct = 26,9)	Negative	Negative	Negative		-	Discharged
Case 2	29	37	Positive (Ct=23.06)	Moderate	Negative	Negative	Negative	-	Negative	-	Discharged
Case 3	34	32	Positive (Ct=34.2)	Severe	Negative	Negative	Negative	-	Negative	-	Hospitalized
Case 4	31	36	Positive (Ct=26.58)	Moderate	Negative	Negative	Negative	Negative	-	-	Discharged
Case 5	39	35	Negative	Moderate	Negative	Negative	Negative	-	Negative	-	Discharged
Case 6	29	39	Positive (Ct=20.67)	Moderate	Negative	Negative	Negative	-	Negative	-	Discharged
Case 7	42	37	Positive (Ct= 32.01)	Critical	Negative	Negative	Negative	-	-	Positive	Discharged
Case 8	39	33	Positive (Ct=26.14)	Moderate	Negative	Negative	Negative	-	-	Negative	Discharged
Case 9	36	28	Positive (Ct=33.92)	Critical	Negative	Negative	Negative	-	-	Negative	Stillbirth
Case 10	37	29	Negative	Critical	Negative	Negative	Negative	Negative	-	-	Hospitalized
Case 11	35	29	Positive (Ct= 33.64)	Severe	Negative	Negative	Negative	-	Negative	-	Hospitalized
Case 12	29	37	Positive (Ct=22.91)	Moderate	Negative	Negative	Negative	-	Negative	-	Discharged
Case 12	29	37	Positive (Ct=22.91)	Moderate	Negative	Negative	Negative	-	Negative	-	Discharged

GA: gestational age; PCR: polymerase chain reaction to detect SARS-CoV-2, indicating the Cycle threshold (Ct).

Regarding maternal comorbidity, we found one case of urosepsis, one of obesity, one of anemia and nine did not have comorbidities. Of the thirteen newborns born between 28 and 39 weeks, three went to the NICU and one died in utero. One 28-week fetal death was reported whose mother had severe disease (COVID-19 pneumonia, *Acinetobacter baumannii* bacterial infection, reactive PB thrombosis). The fetal death had negative PCR for SARS-CoV-2. The neonatal discharge diagnoses were the following: three with jaundice, four with adequate gestational age, one with low birth weight and one large for gestational age.

### Anatomopathological results

**Figure 1:**
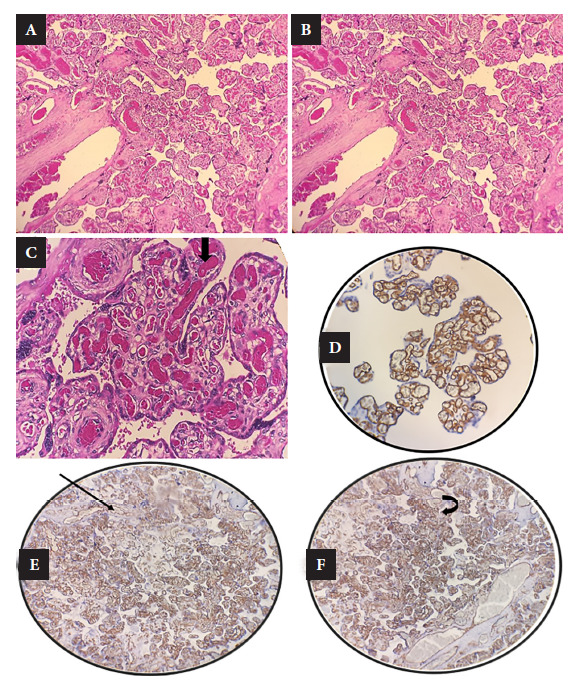
Placental microscopy results. A: numerous blood vessels in the chorionic villi with areas of thrombosis and syncytial knot proliferation. B: chorangiomatosis proliferation of capillaries. C: chorangiomatosis. D: Immunohistochemical staining with CD34. E: CD34 shows staining of capillary vessel walls. F: Immunohistochemistry with positive CD34 shows staining of dilated blood vessels.

Twelve placentas were studied and eleven were by sections of placental parenchyma, umbilical cord and membranes plus one total chorionic-biamniotic twin placenta (with umbilical cords and membranes). There were three cases of placentas with chorangiosis and hypervascularization (25%). We found more than ten capillaries per chorionic villus in chorangiosis cases, the etiology of which is frequent in preeclampsia. We found some foci of mild acute vellitis in 25% of placentas. Only one case had foci of immaturity of some chorionic villi. We also found alterations of the vascular epithelium, mild inflammatory congestion and some capillary vessels with thrombosis and hemorrhage in one case. The acute inflammatory infiltrate was mild in some chorionic villi (Figure 1).

There was one case of stillbirth at 28 weeks gestational age, in which the placental study showed mild intervascular congestion, presence of mild inflammatory infiltrate, focal chorangiosis, presence of intervillous hyaline degeneration and some villous trunks. SARS-CoV-2 infection could not be detected in any of the cases.

There was one case of a symptomatic but COVID-19 negative woman, whose placenta had PCR for SARS-CoV-2 and showed cord with thrombi, acute vellitis and intervellitis in mild amount, intervillous and perivillous hyaline degeneration, congestion and intervillous vascular hemorrhage.

## DISCUSSION

Transmission of SARS-CoV-2 from mother to fetus was not documented. Negative tests were recorded in amniotic fluid and umbilical cord blood; as suggested by Allotey *et al*. and Kirtsman *et al*. ^10^^,^^11^, with a single case of positive PCR in the placenta and a single asymptomatic positive case in a neonate, the overall SARS-CoV-2 positivity rate in newborns born to infected mothers was low (<1%).

Focal chorangiosis and SARS-CoV-2 infection were detected. In our study, three neonates (25%) required intensive care, a figure that differs from another study that reported a mortality rate of 30-50% and an ICU admission rate of approximately 50% in neonates ^13^.

Algarroba ^14^, Penfield *et al*. ^15^^)^ and Pulinx *et al*. ^16^^)^ reported cases in which placental tissue, membranes and amniotic fluid were tested for SARS-CoV-2 with positive results; our study detected only one positive result in placental tissue. On the other hand, six pregnant women presented pneumonia (50%) and required admission to the ICU and about 33% required mechanical ventilation, which is similar to the findings by Dong *et al.*^17^. The rate of severe pneumonia reported was similar to other studies, ranging from 0% to 46%. The increased and higher expression of this receptor on the plasma membrane of angiotestin-converting enzyme 2 (ACE2) upon binding to the viral spike protein in the second trimester of pregnancy would increase the possibility of vertical transmission of the virus ^18^. It is possible that viral infection leads directly to placental disease, or that there is a common underlying cause for both placental lesions and susceptibility to SARS-CoV-2 ^19^.

We found three cases with chronic placental inflammatory process, but none of high grade. Although this represents an overall frequency of 15%, there was only one case of chronic inflammation of the villi (VUE, not gradable). Previous studies include only case reports identifying one chronic inflammatory process and three intervillitis; one of our cases had intervillitis, but it was not confirmed in other described series ^20^.

Our study has some limitations. One of the main limitations is that HNERM is a referral hospital, so the patient characteristics could differ from those at other levels of care; therefore, our results should be extrapolated with caution. Then, the limited number of cases does not allow generalizations to be made. We were unable to perform electron microscopic analysis in order to achieve a more sensitive detection of SARS-CoV-2 in the placenta. However, this is the first study in Peru to evaluate vertical transmission using PCR testing, the gold standard for SARS-CoV-2 detection.

In conclusion, vertical transmission of COVID-19 from mother to fetus is uncommon. Although one case was detected in a neonate, the frequency of vertical transmission was low, suggesting a low incidence of transmission during pregnancy. Further research is needed to better understand the relationship between infection and placental complications during pregnancy.

## References

[1] World Health Organization (2024). WHO COVID-19 dashboard..

[2] Akhtar H, Patel C, Abuelgasim E, Harky A (2020). COVID-19 (SARS-CoV-2) Infection in Pregnancy A Systematic Review. Gynecol Obstet Invest.

[3] Ministerio de Salud (2022). Sala de Situación SE 08 - 2022.

[4] Dávila-Aliaga C, Hinojoza-Pérez R, Espinola-Sánchez M, Torres-Marcos E, Guevara-Ríos E, Espinoza-Vivas Y (2021). Resultados materno-perinatales en gestantes con COVID-19 en un hospital nivel III del Perú. Rev Peru Med Exp Salud Pública.

[5] Capobianco G, Saderi L, Aliberti S, Mondoni M, Piana A, Dessole F (2020). COVID-19 in pregnant women A systematic review and meta-analysis. Eur J Obstet Gynecol Reprod Biol.

[6] Kotlyar AM, Grechukhina O, Chen A, Popkhadze S, Grimshaw A, Tal O (2021). Vertical transmission of coronavirus disease 2019: a systematic review and meta-analysis. Am J Obstet Gynecol.

[7] Yan J, Guo J, Fan C, Juan J, Yu X, Li J (2020). Coronavirus disease 2019 in pregnant women a report based on 116 cases. Am J Obst Gynecol.

[8] Della Gatta AN, Rizzo R, Pilu G, Simonazzi G (2020). Coronavirus disease 2019 during pregnancy a systematic review of reported cases. Am J Obst Gynecol.

[9] Lamouroux A, Attie-Bitach T, Martinovic J, Leruez-Ville M, Ville Y (2020). Evidence for and against vertical transmission for SARS-CoV-2 (COVID-19). Am J Obstet Gynecol.

[10] Allotey J, Chatterjee S, Kew T, Gaetano A, Stallings E, Fernández-García S (2022). SARS-CoV-2 positivity in offspring and timing of mother-to-child transmission living systematic review and meta-analysis. BMJ.

[11] Kirtsman M, Diambomba Y, Poutanen SM, Malinowski AK, Vlachodimitropoulou E, Parks WT (2020). Probable congenital SARS-CoV-2 infection in a neonate born to a woman with active SARS-CoV-2 infection. CMAJ.

[12] Khong TY, Mooney EE, Ariel I, Balmus NCM, Boyd Tk, Brundler MA (2016). Sampling and Definitions of Placental Lesions Amsterdam Placental Workshop Group Consensus Statement. Arch Pathol Lab Med.

[13] Vivanti AJ, Vauloup-Fellous C, Prevot S, Zupan V, Suffee C, Do Cao J (2020). Transplacental transmission of SARS-CoV-2 infection. Nat Commun.

[14] Algarroba AN, Hanna NH, Rekawek P, Vahanian SA, Khullar P, Palaia T (2020). Confirmatory evidence of the visualization of severe acute respiratory syndrome coronavirus 2 invading the human placenta using electron microscopy. Am J Obstet Gynecol.

[15] Penfield CA, Brubaker SG, Limaye MA, Lighter J, Ratner Aj, Thomas KM (2020). Detection of SARS-COV-2 in placental and fetal membrane samples. Am J Obstet Ginecol MFM.

[16] Pulinx B, Kieffer D, Michiels I, Petermans S, Strybol D, Delvaux S (2020). Vertical transmission of SARS-CoV-2 infection and preterm birth. Eur J Microbiol Infect Dis.

[17] Dong L, Tian J, He S, Zhu C, Wang J, Liu C (2020). Possible Vertical Transmission of SARS-CoV-2 From an Infected Mother to Her Newborn. JAMA.

[18] Shanes ED, Mithal LB, Otero S, Azad HA, Miller ES, Goldstein JA (2020). Placental Pathology in COVID-19. Am J Clin Pathol.

[19] Juan J, Gil MM, Rong Z, Zhang Y, Yang H, Poon LC (2020). Effect of coronavirus disease 2019 (COVID-19) on maternal, perinatal and neonatal outcome systematic review. Ultrasound Obstet Gynecol.

[20] Zamaniyan M, Ebadi A, Aghajanpoor S, Rahmani Z, Haghshenas M, Azizi S (2020). Preterm delivery, maternal death, and vertical transmission in a pregnant woman with COVID-19 infection. Prenat Diagn.

