# Supplementary material for: SARS-CoV-2 infection during pregnancy: clinical characteristics and vertical transmission in a referral hospital in Peru
Source: Rev Peru Med Exp Salud Publica. 2024 May 20;41(2):178–84. doi: 10.17843/rpmesp.2024.412.13293 (PMC11300692; doi:10.17843/rpmesp.2024.412.13293)
Supplement: Translation

# Translation: Infección por SARS-CoV-2 en el embarazo: características clínicas y trasmisión vertical en un hospital de referencia de Perú

**Authors:** Claudia Aracelli Urbina-Alvarez, Julio Cesar Sifuentes-Alvarez, Juan Felipe Moreno-Bocanegra, Kevin Vasquez-Sandoval, Lilia Huiza-Espinoza, Mauricio La Rosa-De los Rios, Juan Carlos Gomez De La Torre-Pretell, Claudia Fiorella Barletta-Carrillo

**Affiliations:** 1 ospital Nacional Edgardo Rebagliati Martins, Lima, Perú.; 2 Hospital San Bartolomé, Lima, Perú.; 3 Universidad De Texas Medical Branch, Texas, Estados Unidos.; 4 Universidad Peruana Cayetano Heredia, Lima, Perú.; 5 Laboratorio ROE, Lima, Perú.; 6 Sequence Reference Lab, Lima, Perú.; 7 Universidad Nacional Mayor de San Marcos, Lima, Perú.

**Keywords:** Transmisión vertical de enfermedad infecciosa, COVID-19, Embarazo, Neonato

## Abstract

Se realizó un estudio en el departamento de Ginecología y Obstetricia del Hospital Nacional Edgardo Rebagliati Martins (HNERM) con el objetivo analizar la transmisión vertical del SARS-CoV-2 en mujeres embarazadas con COVID-19. Se incluyeron 12 gestantes que cumplían con los criterios de inclusión. Se realizaron pruebas diagnósticas de PCR en tiempo real (RT-PCR) para SARS-CoV-2 durante la admisión de cada gestante y se recolectaron muestras de placenta para su evaluación anatomopatológica. Los resultados mostraron que la transmisión vertical del virus fue poco común, con una tasa general de positividad baja en los recién nacidos. Aunque el estudio presenta limitaciones, como el número reducido de casos y la falta de análisis con microscopio electrónico, constituye el primer intento en Perú de evaluar la transmisión vertical. Se concluye que se necesita más investigación para comprender mejor la relación entre la infección por la COVID-19 y las complicaciones durante el embarazo.

## INTRODUCCIÓN

Durante la primera semana de febrero del 2022, la pandemia de la COVID-19 continuaba ejerciendo un impacto significativo a nivel mundial, con un total de 490 millones de casos confirmados y más de 5,8 millones de defunciones. El Perú fue uno de los países más afectados por esta enfermedad, con más de 3,3 millones de casos confirmados y casi 207 mil fallecidos ^1^. Diversas investigaciones han evidenciado que la infección por el virus SARS-CoV-2 puede provocar una afectación multisistémica en mujeres embarazadas, con complicaciones que van desde partos prematuros hasta la muerte materna ^2^. En Perú, al igual que en otras partes del mundo, la COVID-19 ha tenido un impacto significativo en la atención de la salud materna. Durante el año 2021, se registró un aumento del 12% en las muertes maternas en comparación con el 2020, y una de cada nueve de estas muertes estuvo relacionada con la COVID-19 ^3^. Las complicaciones más comunes fueron la rotura prematura de membranas y la preeclampsia, mientras que el 14% de los recién nacidos presentaron condiciones como prematuridad, bajo peso al nacer, sepsis y neumonía ^4^.

Durante el embarazo, se producen cambios fisiológicos que convierten a las gestantes en una población de riesgo ^5^^,^^6^. La COVID-19 puede tener graves repercusiones en las mujeres embarazadas. Además, aunque la transmisión vertical del coronavirus es un evento poco común ^7^, están surgiendo problemas relacionados con esta transmisión ^8^. La viremia estuvo presente en el 1% de los pacientes adultos que mostraban síntomas de la enfermedad por coronavirus en el 2019 ^9^. Para determinar si la infección ocurrió de manera congénita o perinatal, se consideran la exposición perinatal, el tipo de parto y el tiempo transcurrido desde el parto hasta el diagnóstico de la infección neonatal. Los recién nacidos con infección por coronavirus suelen ser asintomáticos ^9^.

Existe muy poca evidencia sobre la transmisión intrauterina de la madre al feto o la transmisión intraparto de la madre al recién nacido. En casos de infección materna tardía, se debe considerar la posibilidad de que el recién nacido presente una infección activa y, por ende, esté en riesgo de sufrir complicaciones, así como la posibilidad de que el bebé represente riesgos para los trabajadores de la salud. En este artículo nos centramos en los recién nacidos cuyas madres tuvieron COVID-19 documentado o sospechado en el momento del parto ^6^^,^^10^^,^^11^. Por lo tanto, el objetivo fue analizar la transmisión vertical del SARS-CoV-2 en mujeres embarazadas con COVID-19 en el Hospital Nacional Edgardo Rebagliati Martins (HNERM).

**MENSAJE CLAVE**

Motivación para realizar el estudio: A pesar de la evidencia de una afectación multisistémica en mujeres embarazadas con la COVID-19, existe un vacío de conocimiento sobre la transmisión vertical del virus y sus implicancias en la salud materna y neonatal.

Principales hallazgos: Los resultados del estudio sugieren una baja incidencia de transmisión vertical durante el embarazo, con solo un caso positivo de PCR en la placenta y un neonato asintomático.

Implicancias: Este resultado pueden informar las estrategias de prevención y manejo de la COVID-19 en mujeres embarazadas, así como guiar el desarrollo de políticas sanitarias dirigidas a proteger la salud materna y neonatal durante la pandemia.

## EL ESTUDIO

### Diseño y escenario

El estudio fue descriptivo y se realizó en el departamento de Ginecología y Obstetricia del HNERM entre enero de 2021 y enero de 2022.

### Población

El estudio incluyó a gestantes admitidas en el HNERM que cumplieron los siguientes criterios de inclusión: sospecha clínica y/o radiológica y/o laboratorial de infección por SARS-CoV-2 y alguna indicación para término de gestación por cesárea en el segundo y tercer trimestre. Las participantes firmaron un consentimiento informado. Se excluyeron del estudio a las pacientes que habían tenido un aborto y/o estaban en el primer trimestre de gestación, así como aquellas que no dieron su consentimiento para participar. Doce gestantes cumplieron los criterios de selección.

### Variables de estudio

La variable independiente fue el grado de severidad de la infección por COVID-19 durante el embarazo; los casos se clasificaron según lo propuesto en Wuhan: moderados (neumonía leve sin insuficiencia respiratoria aguda ni respuesta inflamatoria), severos (neumonía con insuficiencia respiratoria aguda, inflamación o hipercoagulabilidad) y críticos (con criterio de intubación y ventilación invasiva, choque o falla multiorgánica).

Las variables del estudio, sus categorías y unidades de medida se muestran en la tabla 1. Los datos se recolectaron en una ficha única por cada paciente en un intervalo de tiempo único para análisis posterior.

**Tabla 1:** Características maternas de pacientes incluidas en el estudio (n=12).

Variable		Unidad de medida
Características sociodemográficas		
	Edad materna	años
	Antecedente de edad gestacional media al parto	semanas
	Mortalidad neonatal	sí/no
Hallazgos clínicos		
	FC	respiraciones/min
	FR	latidos/min
	PAS	mmHg
	PAD	mmHg
	Saturación de oxígeno	%
	Requerimiento de oxígeno	L
	Hallazgos radiológicos de neumonía	sí/no
	Admisión a UCI materna	sí/no
	Uso de ventilación mecánica	sí/no
	Tiempo de enfermedad	días
Hallazgos laboratoriales		
	LDH	U/L
	PCR	U/L
	TGO	U/L
	Recuento de plaquetas	por 1000/UL
	Leucocitos	por 1000/UL
	Creatinina	mg/dL
	Linfocitos	por 1000/UL

FC: frecuencia cardíaca, FR: frecuencia respiratoria, PAS: presión arterial sistólica, PAD: presión arterial diastólica, UCI: unidad de cuidados intensivos, LDH: deshidrogenasa láctica, PCR: proteína C reactiva, TGO: transaminasa oxalacética.

### Fuente de datos/medidas y procedimiento

Durante la admisión de cada gestante se realizaron pruebas diagnósticas de PCR en tiempo real (RT-PCR) para SARS-CoV-2 a partir de muestras de hisopado nasal de la gestante, hisopado nasal del neonato en las primeras 72 horas, hisopado de las membranas placentarias, líquido amniótico y sangre del cordón. Se obtuvo muestras de placenta para una posterior evaluación microscópica.

Para el hisopado de membranas y extracción de sangre de cordón umbilical, los hisopados placentarios (PCR placentaria) se obtuvieron de la superficie amniótica (fetal) después de limpiar la superficie de la sangre materna y fueron recolectados en el espacio entre el amnios y el corion después de la separación manual cuidadosa de las membranas. Para las muestras de sangre de cordón umbilical, se extrajo el plasma y se almacenó a -80 °C hasta su procesamiento.

La prueba de PCR para SARS-CoV-2 fue realizada mediante extracción de ARN de muestras biológicas con reactivo Sbeadex livestock kit (LGC Biosearch Technologies) en plataforma automatizada oKtopure y la detección molecular a través de la amplificación del gen *RdRp* del virus y de *RNAse P* como control interno con el kit Logix Smart SARS-CoV-2 test de Co-Diagnostics Inc en termocicladores Cobas Z 480 (Roche) o CoDx-Box (Biomolecular Systems).

Las placentas fueron fijadas en formalina y embebidas en parafina, se realizaron tinciones con la coloración de hematoxilina/eosina. También se realizó la inmunohistoquímica con CD34.

Respecto a la anatomía patológica, los hallazgos placentarios microscópicos se clasificaron como patología inflamatoria aguda correspondiente a etapas de inflamación materna y fetal con infiltrado de neutrófilos (coriamnionitis histológica y arteritis umbilical); y patología inflamatoria crónica: infiltrado de linfocitos e histiocitos (villitis linfocítica crónica y deciduitis crónica con células plasmáticas). Para la intervellositis crónica, se utilizó el Consenso de Amsterdam ^12^: bajo grado (menor de 10 vellosidades afectadas por foco o vista al microscopio y multifocal si se encuentra en varias láminas); y, alto grado (mayor de 10 vellosidades en cualquier campo, puede ser en parche o difusa, si es mayor del 30% del parénquima afectado).

### Análisis estadístico

Las fichas únicas se trasladaron a una planilla en MS Excel para codificarlas y poder ingresarlas al programa STATA versión 16.0 para Windows para su análisis estadístico. Por tratarse de un estudio descriptivo, se realizó una doble digitación y corrección de errores. En las variables cuantitativas se evaluó la normalidad con la prueba de Shapiro-Wilk y se empleó la media y desviación estándar o mediana y rango intercuartílico, de acuerdo con su distribución.

### Aspectos éticos

Antes de iniciar el estudio se solicitó la firma del consentimiento informado de cada participante. El estudio no presentó riesgos para las pacientes. Se siguieron protocolos estrictos de bioseguridad para la toma, procesamiento y traslado de las muestras. El estudio fue evaluado y aprobado por el Comité de Ética en Investigación Especializado en COVID-19 de Essalud. (Resolución N°42-IETSI-ESSALUD 2020 de fecha 27 marzo 2020) y código PRISA N.° EI00001364.

## HALLAZGOS

### Resultados materno-perinatales

Las características de las participantes en el estudio se presentan en la tabla 2. De las doce gestantes incluidas, once gestaciones fueron simples y una gemelar. Entre los síntomas de las gestantes se encontró una con odinofagia, dos con dolor torácico, dos con aleteo nasal, cuatro con fiebre, siete con disnea y ocho con tos. Nueve dieron resultado positivo a la prueba molecular por PCR para SARS-CoV-2 y tres fueron incluidas debido al cuadro clínico, radiológico y al resultado de prueba antigénica compatible con la COVID-19. Dos casos de infección por SARS-CoV-2 fueron severos y cuatro casos críticos ingresaron a la unidad de cuidados intensivos (UCI). Todas las pacientes fueron sometidas a cesárea. Se tuvo un caso positivo de SARS-CoV-2 en un neonato que fue asintomático (Tabla 3).

**Tabla 2:** Características maternas de pacientes incluidas en el estudio (n=12).

Características	Media (min-max)
Edad materna	33,8 (29-42 años)
Edad gestacional	34,3 (28-39 semanas)
Ingreso a UCI materna	6
Neumonía	6
Ventilación mecánica	4
Tiempo de enfermedad	5,4 (2-15)
LDH	326,2 (183-391)
PCR	504 (11-1735)
TGO	70,5 (28-164)
Recuento de plaquetas	262 (110-936)
Leucocitos	11,1 (5,3-26,0)
Creatinina	0,5 (0,4-0,8)
Linfocitos	1,4 (0,5-4,0)
FC	93 (74-118)
FR	22 (17-28)
PAS	113 (100-130)
PAD	67,5 (60-70)
Saturación de oxígeno	90,4 (63-98)
Requerimiento de oxígeno	12,28 (3-16)

UCI: unidad de cuidados intensivos; LDH: deshidrogenasa láctica; PCR: proteína C reactiva; TGO: transaminasa oxalacética; PAS: presión arterial sistólica; PAD: presión arterial diastólica.

**Tabla 3:** Características de cada caso incluido.

Caso	Edad de la madre	EG	PCR materno	Severidad de la infección materna	PCR placenta	PCR líquido	PCR cordón	Día de vida 1	Día de vida 2	Día de vida 3	Desenlace neonatal
Caso 1	31	37	Negativo	Crítica	Positivo (Ct = 26,9)	Negativo	Negativo	Negativo		-	Alta
Caso 2	29	37	Positivo (Ct=23,06)	Moderada	Negativo	Negativo	Negativo	-	Negativo	-	Alta
Caso 3	34	32	Positivo (Ct=34,2)	Severa	Negativo	Negativo	Negativo	-	Negativo	-	Internado
Caso 4	31	36	Positivo (Ct=26,58)	Moderada	Negativo	Negativo	Negativo	Negativo	-	-	Alta
Caso 5	39	35	Negativo	Moderada	Negativo	Negativo	Negativo	-	Negativo	-	Alta
Caso 6	29	39	Positivo (Ct=20,67)	Moderada	Negativo	Negativo	Negativo	-	Negativo	-	Alta
Caso 7	42	37	Positivo (Ct= 32,01)	Crítica	Negativo	Negativo	Negativo	-	-	Positivo	Alta
Caso 8	39	33	Positivo (Ct=26,14)	Moderada	Negativo	Negativo	Negativo	-	-	Negativo	Alta
Caso 9	36	28	Positivo (Ct=33,92)	Crítica	Negativo	Negativo	Negativo	-	-	Negativo	Óbito
Caso 10	37	29	Negativo	Crítica	Negativo	Negativo	Negativo	Negativo	-	-	Internado
Caso 11	35	29	Positivo (Ct= 33,64)	Severa	Negativo	Negativo	Negativo	-	Negativo	-	Internado
Caso 12	29	37	Positivo (Ct=22,91)	Moderada	Negativo	Negativo	Negativo	-	Negativo	-	Alta
Caso 12	29	37	Positivo (Ct=22,91)	Moderada	Negativo	Negativo	Negativo	-	Negativo	-	Alta

EG: edad gestacional; PCR: reacción en cadena de la polimerasa para detectar SARS-CoV-2, que indica el Cycle threshold (Ct) o ciclo umbral.

Respecto a la comorbilidad materna se encontró un caso de urosepsis, uno de obesidad, uno de anemia y nueve sin comorbilidad. De los trece recién nacidos entre las 28 y 39 semanas, tres fueron a UCI y uno falleció intraútero. Se informó de una muerte fetal de 28 semanas cuya madre tuvo enfermedad grave (neumonía por COVID-19, bacteriana por *Acinetobacter baumannii*, trombositosis PB reactiva). El óbito fetal tuvo PCR negativa para SARS-CoV-2. Los diagnósticos de egreso neonatal fueron, tres con ictericia, cuatro con adecuada edad gestacional, uno con bajo peso al nacer y uno grande para la edad gestacional.

### Resultados anatomopatológicos

Se estudiaron doce placentas y once fueron por secciones de parénquima placentario, cordón umbilical y membranas además una placenta gemelar total coriónica-biamniótica (con cordones umbilicales y membranas). Hubo tres casos de placentas con hallazgos de corangiosis e hipervascularización (25%). En corangiosis encontramos más de diez capilares por vellosidad corial y cuya etiología es frecuente en preeclampsia. En un 25% de las placentas se encontró algunos focos de vellitis aguda leve. En un solo caso se encontraron focos de inmadurez de algunas vellosidades coriales. Los hallazgos encontrados son de alteraciones del epitelio vascular, congestión inflamatoria leve y algunos vasos capilares con trombosis y hemorragia en un caso. El infiltrado inflamatorio agudo fue leve en algunas vellosidades coriales (Figura 1).

**Figura 1:** Resultados de la microscopía de placenta. A: numerosos vasos sanguíneos en las vellosidades coriales con zonas de trombosis y proliferación nudos sincitiales. B: corangiomatosis proliferación de capilares. C: corangiosis. D: tinción de Inmunohistoquímica con CD34. E: CD34 nos demuestra tinción de paredes de vasos capilares. F: inmunohistoquímica con CD34 positivo muestra tinción de vasos sanguíneos dilatados

Se presentó un caso de natimuerto de 28 semanas de edad gestacional en cuyo estudio de placenta mostró congestión intervascular leve, presencia de infiltrado inflamatorio leve, corangiosis focal, presencia de degeneración hialina intervellosa y de algunos troncos vellosos. La infección por SARS-CoV-2 no se pudo detectar en ninguno de los casos.

Hubo un caso de una mujer sintomática pero negativa a la prueba de COVID-19, cuya placenta tuvo PCR para SARS-CoV-2 y mostró cordón con presencia de trombos, presencia de vellitis e intervellitis aguda en leve cantidad, presencia de degeneración hialina intervellosa y perivellosa, congestion y hemorragia vascular intervellosa.

## DISCUSIÓN

No se logró documentar la transmisión del SARS-CoV-2 de la madre al feto. Se registraron pruebas negativas en líquido amniótico y sangre del cordón umbilical; como sugieren Allotey *et al.* y Kirtsman *et al*. ^10^^,^^11^, al registrarse un único caso de PCR positiva en la placenta y un solo caso positivo asintomático en un neonato, la tasa general de positividad del SARS-CoV-2 en recién nacidos de madres infectadas fue baja (<1%).

Se detectó la presencia de corangiosis focal e infección por SARS-CoV-2. En nuestro estudio, tres casos (25%) de neonatos requirieron cuidados intensivos, una cifra que difiere de otro estudio que reportó una tasa de mortalidad del 30-50% y una tasa de ingreso en la UCI de aproximadamente el 50% en neonatos ^13^.

Algarroba ^(^^14^, Penfield *et al.*^15^^)^ y Pulinx *et al.*^16^ informaron casos en los que se analizó tejido placentario, membranas y líquido amniótico para SARS-CoV-2, con resultados positivos; nuestro estudio detectó solo un resultado positivo en el tejido placentario. De otro lado, seis gestantes presentaron neumonía (50%) y requirieron ingreso en la UCI y cerca del 33% necesitaron ventilación mecánica, concidiendo con los hallazgos de Dong *et al*. ^17^. La tasa de neumonía grave reportada fue similar en otros estudios, variando entre el 0% y el 46%. El aumento y mayor expresión de este receptor en la membrana plasmática de la enzima convertidora de la angioestina 2 (ACE2) al unirse a la proteina viral *spike* en el segundo trimestre del embarazo aumentaría la posibilidad de transmisión vertical del virus ^18^. Es posible que la infección viral conduzca directamente a la patología placentaria, o que exista una causa subyacente común tanto para las lesiones placentarias como para la susceptibilidad al SARS-CoV-2 ^(^^19^.

En nuestra investigación, tres casos presentaron proceso inflamatorio placentario crónico, pero ninguno de alto grado. Aunque esto representa una frecuencia general del 15%, solo hubo un caso de inflamación crónica de las vellosidades (VUE, no graduable). En la literatura publicada solo existen reportes de casos identificando un proceso inflamatorio crónico y tres intervellositis; en uno de nuestros casos se observó intervellositis, pero no se confirmó en otras series descritas ^20^.

Algunas limitaciones del presente estudio deben tomarse en cuenta. Primero, el HNERM es un hospital de referencia, por lo que los pacientes podrían ser diferentes a los de otros niveles de atención, debido a esto se debe tener cuidado al extrapolar los resultados de este estudio. Segundo, el limitado número de casos de estudio no permite hacer generalizaciones. Tercero, no pudimos realizar análisis con microscopio electrónico para poder realizar una detección más sensible del SARS-CoV-2 en la placenta. Sin embargo, este es el primer estudio realizado en Perú que evalúa la trasmisión vertical utilizando la prueba de PCR, estándar de oro para detección de SARS-CoV-2.

En conclusión, la transmisión vertical de la COVID-19 de la madre al feto es poco común. Aunque se detectó un caso en un neonato la frecuencia de transmisión vertical fue baja, lo que sugiere una baja incidencia de transmisión durante el embarazo. Se necesita más investigaciones para comprender mejor la relación entre la infección y las complicaciones placentarias durante el embarazo.
